# Feasibility of A Multi‐Pronged, Multi‐Sensor Cognitive Aging Protocol in a diversified Cohort: Results from the Einstein Aging Study

**DOI:** 10.1002/alz.092979

**Published:** 2025-01-09

**Authors:** Nelson A. Roque, Martin J. Sliwinski, Carol A. Derby, Richard B. Lipton, Orfeu M. Buxton, Mindy J. Katz

**Affiliations:** ^1^ The Pennsylvania State University, University Park, PA USA; ^2^ Pennsylvania State University, State College, PA USA; ^3^ Department of Neurology, and Department of Epidemiology and Population Health, Albert Einstein College of Medicine, Bronx, NY USA; ^4^ Department of Neurology, Albert Einstein College of Medicine, Bronx, NY USA; ^5^ Pennsylvania State University, University Park, PA USA; ^6^ Albert Einstein College of Medicine, Bronx, NY USA

## Abstract

**Background:**

Older adults, especially from diverse populations, are often presumed to be unwilling or unable to participate in mobile health (mHealth) studies. Since 2017, the Einstein Aging Study (EAS) participants have been instructed on and actively supported in completing comprehensive and intensive longitudinal assessments, harnessing the capabilities of smartphones and wearable devices. Notably, since early 2023, the EAS has expanded its scope by incorporating the gathering of data from multiple wearable sensors.

**Method:**

In addition to clinic‐based neuropsychological testing, neurological exams and blood draws, the main protocol of the Einstein Aging Study asks participants to complete 14‐days of app‐based surveys and ambulatory cognitive assessments five times per day (i.e., upon wake‐up, at bedtime, and three notified assessments in between). Participants are also wearing sensors to collect data on air quality (via Atmotube Pro) and monitoring sleep, physical activity, and sedentary behavior (via Actiwatch, Actipal). We present compliance data for our new Ecological Momentary Assessment protocol.

**Result:**

There are 112 total EAS participants (mean age = 80.87, SD= 6.06; % Female = 71.2; % non‐Hispanic Whites = 38.74%, % non‐Hispanic Blacks = 49.55%, % Hispanics = 11.71%). Overall, across our new ambulatory data collection protocol (i.e., data collected after yearly clinic visit on study smartphones and wearables), the EAS has robust compliance, with median total minutes of daily surveys ∼47 minutes. Specifically, for the Ecological Momentary Assessment (EMA) protocol delivered on study smartphones, 85% of participants completed 11 or more days of wake‐up surveys (self‐initiated), 79% of the first notified survey, 80% of the second, 77% of the third, and 82% completed the bedtime surveys (self‐initiated). There were no significant differences by race or ethnicity in adherence (one‐way ANOVA, all p's > 0.05).

**Conclusion:**

These preliminary data for our longitudinal protocol provide continuing evidence of feasibility that older adults from diverse populations are adherent to intensive longitudinal data collection procedures, including when involving numerous wearable sensors. Future research may explore the influence or perturbations of EMA adherence when adding additional participant burden to a protocol (e.g., adding a continuous glucose monitor (CGM) sensor).

